# Avoiding the Peak of Inflated Expectations: Common Misconceptions in Population Data Research.

**DOI:** 10.23889/ijpds.v7i3.1797

**Published:** 2022-08-25

**Authors:** Peter Christen, Rainer Schnell

**Affiliations:** 1 School of Computing, The Australian National University; 2 University Duisburg-Essen

## Objectives

Databases covering full populations are increasingly used for research studies. Their massive size is often mistaken as a guarantee for valid inferences on the population of interest. However, population data have characteristics that make them challenging to use. We discuss misconceptions about how population data were captured, processed, and linked.

## Approach

We define population data as data about people at the level of a population. The focus on populations is important, as it refers to the scale and complexity of such data, which make manual processing and data quality assessment challenging. Personal data include quasi-identifiers such as names and addresses, as well as microdata such as people’s medical details.

Little consideration has been given to how assumptions about population data can influence the outcomes of a research study. Only few publications describe experiences or challenges when dealing with population data. Many of the misconceptions we discuss are therefore drawn from our experiences over decades working with real-world population databases in collaborations with both private and public sector organisations.

## Results

We identified 32 misconceptions about population data, 21 due to how data are captured (among them “a database contains all individuals in a population”, “records in a population database always refer to real people”, “data definitions are unambiguous”, and “missing data have no meaning”); four due to data processing (including “data processing is always correct” and “metadata are correct, complete, and up-to-date”); and seven due to data linkage (such as “a linked data set corresponds to an actual population”, “a linked data set is unbiased”, and “linkage error rates are independent of database size”).

## Conclusion

Due to misconceptions like those we have identified, careful consideration is needed when personal data at the level of populations are used for research studies. There are no (simple) technical solutions to detect and correct many of these misconceptions; heightened awareness is required by anybody working with population data. We will provide recommendations to help recognise and overcome such misconceptions.

